# Case Report: Potential Applications of Using a 3D-Ring CZT SPECT for Lymphoscintigraphic Exploration of Lower Limb Edema

**DOI:** 10.3389/fmed.2022.866541

**Published:** 2022-03-22

**Authors:** Hélène Besse, Matthieu Bailly

**Affiliations:** Nuclear Medicine Department, CHR d'Orléans, Orléans, France

**Keywords:** lymphoscintigraphy, SPECT/CT, 3D-ring CZT, limb edema, scintigraphy and whole-body imaging

## Abstract

Lymphoscintigraphy is still considered the gold standard imaging modality for diagnosing lymphedema, due to ineffective lymphatic transport resulting in edema and skin damage. However, protocol variability and poor image resolution can make the interpretation challenging. Up to now, 99 mTc-labeled colloid lymphatic travel is monitored with dual-head cameras, but single-photon emission CT (SPECT) has proved its interest. Here, we present the case of a 59-year-old-man with bilateral asymmetric lower limb edema which was explored using dual-head and new 3D-ring cadmium -zinc-telluride (CZT) SPECT cameras, confirming bilateral lower limb lymphatic dysfunction. In line with other recently published reports, this case report promotes the use of SPECT/CT in the lymphoscintigraphic exploration of lower limb edema. The recognition of the clinicopathologic features of lower limb edema is required to prevent missed diagnoses, such as compressive disease, tumors, etc., as well as to better influence the management of patients.

## Introduction

Lymphoedema is a chronic and evolving disease, caused by an insufficiency of lymph circulation that can cause massive morbidity and takes many forms depending on its origin and localization. Though the diagnosis is based on clinical findings, the lymphoscintigraphic investigation of the superficial lymphatic system remains the gold standard for the diagnosis of lower limb lymphedema (1), especially in the clinically less apparent early stages of lymphatic disease (2). This examination is performed with subcutaneously injected 99mTc-labeled colloids, thus reliably providing information about lymph transport. However, the lack of anatomical localization or information such as fibrotic tissues can be considered a potential limitation (3).

Single-photon emission CT (SPECT)/CT combines functional and anatomic information into fused imaging and creates a more powerful diagnostic tool than the separate SPECT and CT. Few studies focused on the added value of SPECT/CT in lymphoscintigraphy to specify the anatomical correlation of lymphatic disorders (4–6).

New 3D-ring cadmium -zinc-telluride (CZT) SPECT/CT systems are now available [Veriton (Spectrum Dynamics Medical) and StarGuide (GE Healthcare)] and allow fast 3D SPECT/CT acquisitions, leading to PET-like utilization (7). To our knowledge, lymphoscintigraphic explorations have never been reported using such cameras.

## Case Report

**Figure 1:**
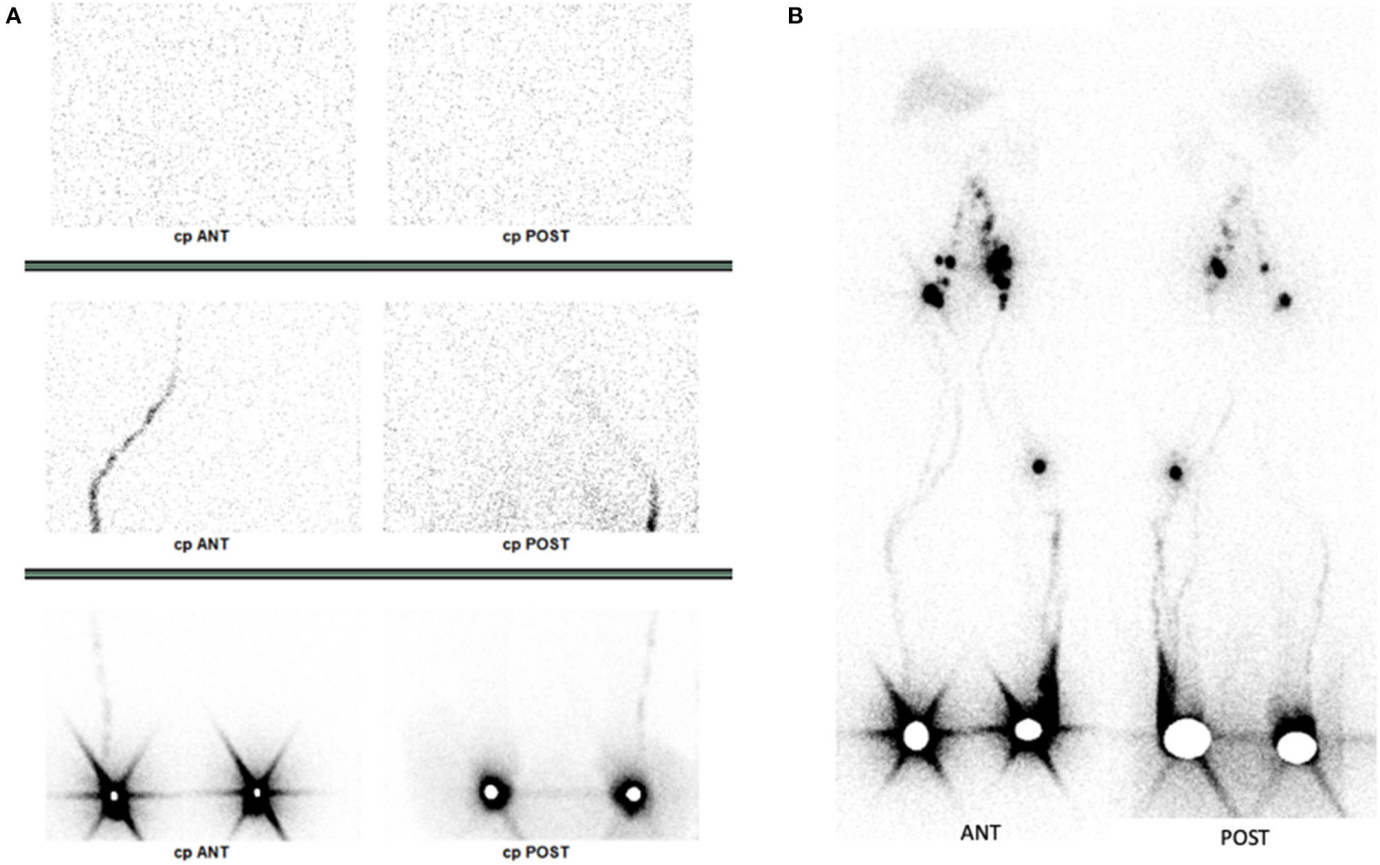
Dual-head conventional camera results. Early anterior and posterior static planar images **(A)**, late anterior and posterior whole-body planar images **(B)**.

**Figure 2:**
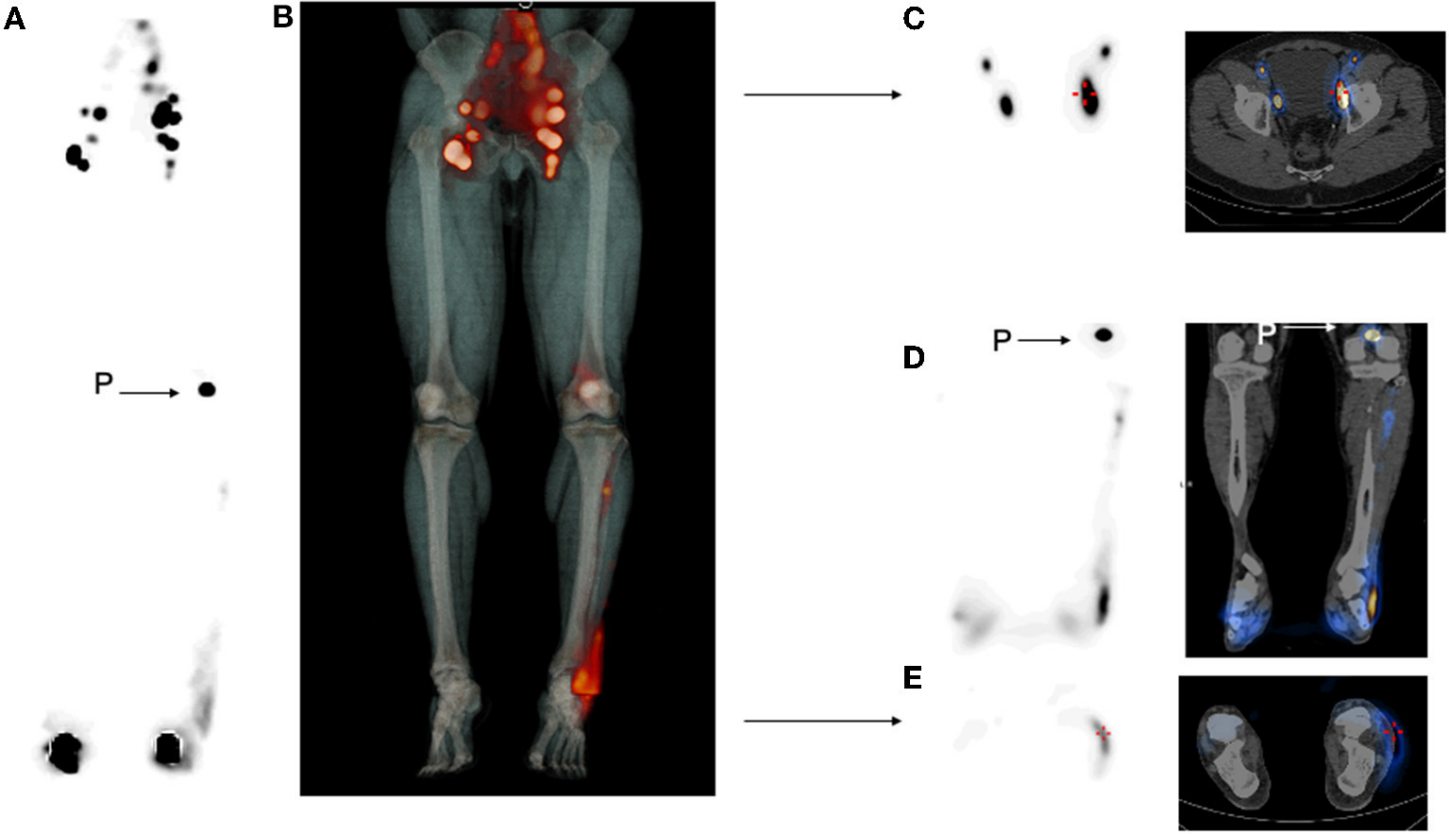
3D-ring cadmium -zinc-telluride single-photon emission CT (CZT SPECT) images. Maximum intensity projection (MIP) anterior view (SPECT only **A**, fused SPECT/CT **B**). **(C)** Axial SPECT and SPECT/CT on the pelvic region. **(D)** Coronal SPECT and SPECT/CT on the leg. **(E)** Axial SPECT and SPECT/CT on the ankles. P with arrow shows the popliteal node. All SPECT images displayed are native non-corrected images.

**Figure 3:**
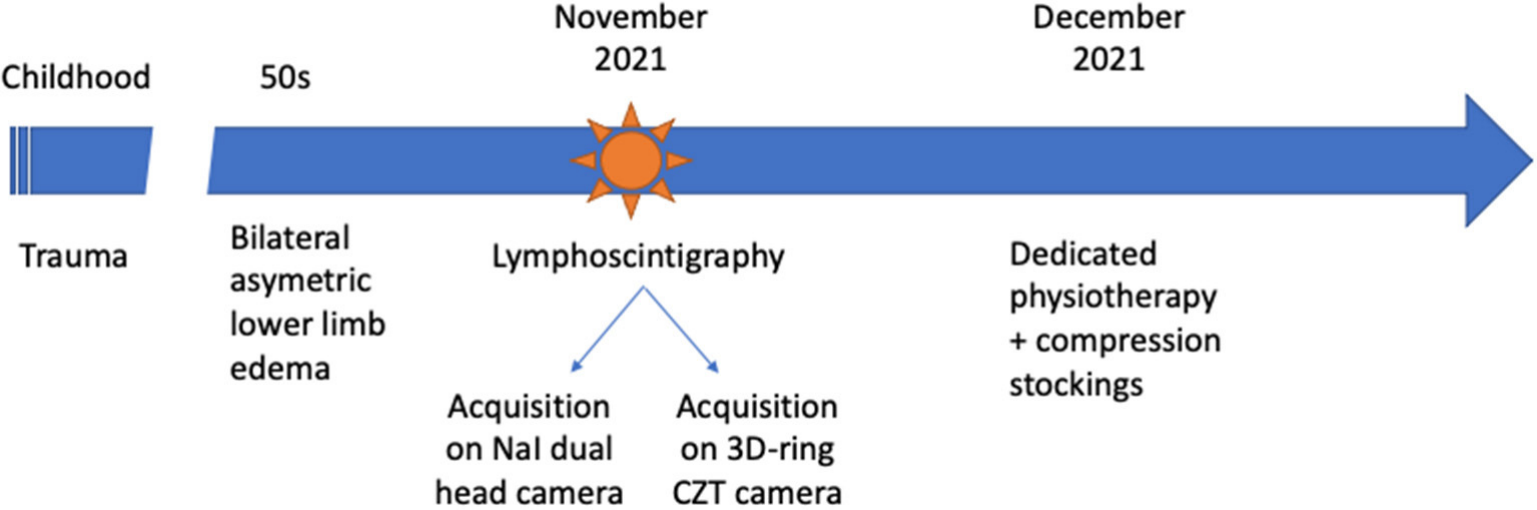
Timeline of diagnosis and intervention for the patient.

We report the case of a 59-year-old-man with a history of bilateral asymmetric lower limb edema. He reported a family history of limb edema and an old trauma in their childhood. On the early planar images after subcutaneous injection in the feet of 114 and 110 MBq 99 mTc-labeled colloids, little migration of the radiopharmaceutical was observed on the right calf and thigh, without any inguinal node seen. No migration was visualized on the left side (Figure 1A). Then, 4 h after injection, late images were acquired both on a dual-head NaI SPECT/CT system (GE Healthcare, Haifa, Israel) with a planar exploration lasting for 12 min (Figure 1B), and on the StarGuide™ 3D-ring CZT SPECT/CT system (GE Healthcare, Haifa, Israel) equipped with 12 swiveling high-resolution CZT detectors. Afterward, 4-bed positions of 3-min uniform SPECT were acquired resulting in 12 min duration SPECT, as well as 40 s of motion and CT (CTDI 2.65 mGy; DLP 312 mGy.cm) (Figure 2). SPECT/CT was only acquired on the new system. On the right side, a single lymphatic network was visualized at the level of the right calf, bifid at the level of the right inner thigh, without recruitment of the deep lymphatic network. Minimal extravasation of the radiopharmaceutical was found in the soft tissues of the ankle, as well as hypoplasia of some pelvic and lumbo-aortic nodes compared to the left side. On the left limb, a sparse lymphatic network was visualized, with collaterals and recruitment of the deep lymphatic network and visualization of a popliteal node, normal inguinal, and pelvic nodes. Dermal stasis with clear extravasation of the radiopharmaceutical at the lateral side of the foot and the ankle is seen on the planar images, but better individualized on SPECT/CT. SPECT/CT also confirmed the recruitment of a left popliteal lymph node network (**P** arrow) and global inguinal and right lumbo-aortic hypoplasia without compressive cause on CT. These results confirmed bilateral lower limb lymphatic dysfunction. Appropriate physiotherapy and compression stockings were prescribed by the referring physician (Figure 3). The patient began to feel clinical improvement after 3 weeks. Regarding the lymphoscintigraphic acquisitions, the patient did not report any trouble or discomfort when using the new 3D-ring CZT SPECT system.

## Discussion

Lymphatic dysfunction may be primary (congenital, praecox, or tarda) or secondary: due to filariasis (8), post-trauma or surgery (9), post-radiation therapy (10), cancer, thyroid disease, obesity, and chronic venous insufficiency. Typical patterns of upper and lower limb lymphedema have been previously described using planar images with dual-head systems (11). Few studies have reported the added value of SPECT/CT to specify the anatomical correlation of lymphatic disorders (2, 4–6).

Different surgical approaches to lymphedema can be considered. Microsurgical treatment of lymphedema, which includes anastomosis between lymphatic vessels and veins or between lymph nodes and veins, requires the identification of functional lymphatic vessels and/or lymph nodes under areas of dermal backflow. Thus, SPECT/CT might be of particular interest (12). Another surgical procedure, liposuction, is used to reduce excess fat deposition in the lymphedematous limb. In this technique, SPECT/CT could be used before surgery to assess the tissue changes related to the lymph stasis, and it could also be used after surgery to understand the functional state of the lymphatic system despite this surgical resection (13). Besides surgery, dedicated physiotherapy with compression stockings is often prescribed which was similar to our patient. SPECT/CT can also guide physical therapists to apply higher pressures in areas with collaterals during their manual maneuvers and/or use of compression system devices (5).

Though the use of SPECT/CT has been described, due to technical limitations, the previous studies only reported 1 or 2 fields of view SPECT/CT (4, 5). The new 3D-ring CZT SPECT camera allows full SPECT acquisitions, using CZT technology. Our case report shows a full pelvic and limbs SPECT/CT for lymphoscintigraphy, acquired in the same duration as the whole-body planar on the conventional dual-head camera. Considering the whole traditional protocol on a dual-head camera, this all-in-one full SPECT acquisition resulted in a time-reduction of more than 50%, including whole body, statics, and mandatory SPECT/CT. The StarGuide SPECT system can acquire native SPECT images, without performing CT. In this observation, we chose to perform a whole CT to have anatomical details of pelvic, popliteal, and ankle regions. Another option could have been to acquire partial CT volume, for example on the popliteal region, to reduce CT exposure, especially in younger patients. Up to now, two major devices rely on 3D-ring CZT design: Veriton SPECT (Spectrum Dynamics Medical) and StarGuide SPECT (GE Healthcare). A recent study using Veriton SPECT found that these new cameras are reliable and that there is no change in the subjective patient experience (14). Regarding the physical performances, a recent study demonstrated that the Veriton CZT camera has superior sensitivity, higher energy resolution, and better image contrast than the conventional SPECT camera, whereas spatial resolution remains similar (15). To our knowledge, this case is the first to report the use of this new generation of SPECT/CT systems for lymphoscintigraphic explorations. New developments in these systems also allow dynamic acquisitions; thus, the next step could be the early-stage dynamic acquisition of lymphoscintigraphy on these 3D-ring CZT SPECT/CT systems, allowing a 4D lymphoscintigraphic exploration, with the anatomical data of CT.

## Conclusion

Our results confirmed that the use of SPECT/CT in lymphoscintigraphic investigations of lymphedema could be helpful and could be quickly available thanks to 3D-ring CZT cameras for the full exploration of both the pelvic region and limbs. The next step could be the acquisition of early 4D-dynamic SPECT with anatomical CT.

## Data Availability Statement

The raw data supporting the conclusions of this article will be made available by the authors, without undue reservation.

## Ethics Statement

The studies involving human participants were reviewed and approved by CPP Nord Ouest IV; ClinicalTrials.gov Identifier: NCT04704349. The patients/participants provided their written informed consent to participate in this study.

## Author Contributions

HB collected the data and interpreted the diagnosis. MB analyzed the literature and wrote the manuscript. All authors contributed to manuscript revision, and read and approved the submitted version.

## Conflict of Interest

MB received honoraria for StarGuide lectures. The remaining author declares that this study received funding from GE Healthcare (technical and financial support paid to the institution). The funder was not involved in the study design, collection, analysis, interpretation of data, the writing of this article or the decision to submit it for publication.

## Publisher's Note

All claims expressed in this article are solely those of the authors and do not necessarily represent those of their affiliated organizations, or those of the publisher, the editors and the reviewers. Any product that may be evaluated in this article, or claim that may be made by its manufacturer, is not guaranteed or endorsed by the publisher.
